# Evaluating implementation of delirium screening in acute care. A qualitative study

**DOI:** 10.1002/alz70858_097016

**Published:** 2025-12-24

**Authors:** Chong Jing Ng, Natalie Jian‐Ting Wee, Philip Lin Kiat Yap, Chin Yee Cheong

**Affiliations:** ^1^ Khoo Teck Puat Hospital, Singapore, Singapore, Singapore; ^2^ Khoo Teck Puat Hospital, Singapore, Singapore; ^3^ Khoo Teck Puat Hospital, Singapore, United Kingdom

## Abstract

**Background:**

Delirium affects an estimated 10‐30% of older adults in acute hospitals, yet delirium often goes unrecognised during admission. Identifying delirium early is thus an important element of care optimisation. We aim to evaluate the barriers and facilitators to delirium screening in acute care from a nursing perspective, as nurses are often the first line of contact with patients.

**Methodology:**

112 acute care nurses practicing in geriatric wards participated in this 2‐staged study. We invited a subset of these nurses to participate in our qualitative evaluation through purposive sampling. 10 nurses ranging from staff nurse grade to advanced practice nurse were interviewed using a semi‐structured interview question based on the Theoretical Domain Framework. Two independent researchers then analysed it using Framework Analysis. A consensus approach was utilised for transcript coding before producing the final results. We identified domains relevant to behaviour changes: 1) Relatively high frequency of domains, 2) Evidence of strong factors that may impact behaviours, 3) Amenable to interventions.

**Results:**

We identified eight theoretical domains that are crucial to the delirium screening implementation. The domains include *Knowledge, Skills, Memory/Attention/Decision Process, Social/Professional Role and Identity, Social Influences, Environmental Context and Resources, Beliefs about Capabilities, and Beliefs about Consequences*. For example, *Knowledge*, a comprehensive understanding of delirium and the screening process were deemed essential for effective practice. *Skills* emerged as a significant barrier, with many nurses highlighting the lack of structured training as a challenge. *Environment Context and Resources*, such as high workloads, also posed significant barriers to routine screening. Conversely, multidisciplinary roles (*Social/Professional Role and Identity*) and colleagues’ perceptions (*Social Influences*) facilitated participants behaviours in carrying out delirium screening.

**Conclusions:**

The use of the TDF domain framework is in keeping with the MRC recommendation of incorporating a theoretical base when developing designs to change behaviours. This study offers a detailed understanding of the key factors influencing the implementation of delirium screening in acute care. Potential interventions include organising workshop/teaching sessions, work‐based discussion, workflow on delirium screening and use of EMR to facilitate compliance. These insights are essential for developing targeted interventions to enhance screening practices and ultimately improve patient outcomes.

